# Comparison of charges and resource use associated with saxagliptin and sitagliptin

**DOI:** 10.1186/s13561-016-0104-8

**Published:** 2016-07-07

**Authors:** Varun Vaidya, Keyuri Adhikari, Jack Sheehan, Iftekhar Kalsekar

**Affiliations:** 1 Health Outcomes and Socioeconomic Sciences, College of Pharmacy and Pharmaceutical Sciences, University of Toledo, Health Science Campus, Mail Stop # 1013, 3000 Arlington Ave., Toledo, OH 43614 USA; 2 Novartis Pharmaceuticals, Hyderabad, India; 3 Health Economics and Outcomes Research, AstraZeneca, Fort Washington, PA 19034 USA; 4 Health Informatics- Medical Devices, Johnson & Johnson, New York, NY USA

## Abstract

**Objectives:**

Saxagliptin and sitagliptin are two commonly used dipeptidyl peptidase-4 (DPP-4) inhibitors. Little is known about their comparative effectiveness in the real world, particularly their impact on cost and resources use. The objective of this study was to analyze charges and resource use associated with saxagliptin and sitagliptin to understand the impact of these DPP-4 inhibitor treatment options in a real-world setting.

**Methods:**

This was a retrospective, new-user study approved by the Institutional Review Board at the University of Toledo. Data were collected from a US insurance claims dataset (OptumInsight) for patients newly initiating treatment with saxagliptin or sitagliptin between January 1, 2010 and December 31, 2011. ICD-9 code 250 was used to identify patients with T2D. Overall and diabetes-related medical and pharmacy charges were observed. Inpatient hospitalizations were also compared. Propensity score matching was used to balance the cohorts of patients prescribed saxagliptin and sitagliptin. Appropriate univariate statistical tests were applied to the propensity-matched sample to examine differences in resource utilization outcomes. Statistical significance was evaluated at *P* < 0.05.

**Result:**

After the propensity score matching, each cohort included 7711 patients. Saxagliptin treatment was associated with lower overall charges ($13,292 vs $14,032; *P* = 0.0023) and overall medical charges ($9,540 vs $10,296; *P* = 0.0024) during the 6-month follow-up period compared with sitagliptin treatment. No significant differences were observed in the overall pharmacy charges ($3,751 vs $3,753; *P* = 0.6937) and the diabetes-related charges ($5,141 vs $5,232; *P* = 0.2957). All-cause and diabetes-related inpatient hospitalization rates were significantly lower with saxagliptin treatment (*p* = 0.0001 and *p* = 0.0019, respectively). All-caused inpatient charges were also significantly lower with saxagliptin ($2,917.26 vs $3445.89; *P* < 0.0001).

**Conclusion:**

Compared with patients initiating sitagliptin treatment, patients initiating saxagliptin treatment reported lower overall and medical charges and lower overall and diabetes-related hospitalization rates. These findings may aid payers in managing patients with T2D.

## Background

Type 2 diabetes mellitus (T2D) accounts for 90–95 % of US adult cases of diabetes [1]. In 2012, the burden of diagnosed diabetes was estimated to be $245 billion, which included $176 billion in direct healthcare expenditures and $69 billion in lost productivity [2]. Biguanides (eg, metformin) are the initial pharmacotherapy of choice for T2D [3–5]. However, patients, particularly those with higher baseline glycated hemoglobin (HbA_1c_) values, may not achieve their glycemic goals on metformin despite titration to the maximum tolerated dose, and therefore may require additional medication [6–8]. As outlined in position statements and society algorithms from the American Diabetes Association/European Association for the Study of Diabetes, the American Association of Clinical Endocrinologists, and the American College of Physicians, disease progression leads to a need for additional antihyperglycemic agents to maintain or improve blood glucose control [9–11]. One such class of antihyperglycemic agents recommended to manage T2D is the dipeptidyl peptidase-4 (DPP-4) inhibitors. DPP-4 is an enzyme that degrades the incretins glucagon-like peptide-1 (GLP-1) and glucose-dependent insulinotropic polypeptide (GIP) [12]. These endogenous polypeptides are released in response to nutrient intake to mediate glucose-dependent insulin secretion. DPP-4 inhibition prevents the inactivation of GLP-1 and GIP, which increases levels of active GLP-1 and GIP. This increase in levels of these active incretins is associated with increased insulin secretion (GLP-1 and GIP) and reduction in glucagon secretion (GLP-1), thereby lowering glucose levels. Also, since the biologic effects of GIP and GLP-1 are glucose-dependent, the risk of hypoglycemia is minimized [13]. The DPP-4 inhibitors approved by the US FDA are sitagliptin (Januvia®, Merck Sharp & Dohme Corp., Whitehouse Station, NJ), saxagliptin (Onglyza®, AstraZeneca, Wilmington, DE), linagliptin (Tradjenta®, Boehringer Ingelheim, Ridgefield, CT), and alogliptin (Nesina®, Takeda Pharmaceuticals America, Inc., Deerfield, IL). Of these, saxagliptin and sitagliptin are the two DDP-4 inhibitors that have been approved and on the market long enough to provide sufficient claim data for cost comparison [14].

Although both saxagliptin and sitagliptin are FDA approved and have established their efficacy through clinical trials [15], little is known about their comparative effectiveness in the real world; more research is needed to measure the impact on cost and resources use [16]. Understanding the impact of these DPP-4 inhibitor treatment options on real-world utilization and charges may assist payers in managing these patients. Thus, the objective of this study was to compare charges and resource use during the 6 months following treatment initiation with saxagliptin or sitagliptin.

## Methods

### Study design

This was a retrospective cohort study that used data from a US insurance claims dataset (OptumInsight) for patients newly initiating treatment with saxagliptin or sitagliptin between January 1, 2010, and December 31, 2011. OptumInsight is a proprietry administrative claims database. It contains both medical and pharmacy claims of more than 40 million beneficiaries from both commercial and public (traditional Medicare and Medicare Advantage) insurances in 50 states. Data from outpatient pharmacy claims included National Drug Codes (NDC) for dispensed medications, quantity dispensed, drug strength, days’ supply, and health plan and patient costs. Medical claims from facilities and providers included International Classification of Diseases, Ninth Revision, Clinical Modification (ICD-9-CM) diagnosis codes, ICD-9 procedure codes, Current Procedural Terminology (CPT) procedure codes, Healthcare Common Procedure Coding System (HCPCS) procedure codes, site of service codes, revenue center codes, provider specialty codes, and health plan and patient costs. While processing data it was made sure that all techniques used to access data are compliant with the Health Insurance Portability and Accountability Act of 1996, and no identifiable protected health information was extracted during the course of the study. The study was approved by the Institutional Review Board at the University at Toledo.

### Patient selection

Each patient included in this study was ≥18 years of age and diagnosed with T2D. To identify patients with T2D, primary or secondary ICD-9 CM codes 250.x0 or 250.x2 were used.

It was also required that patients have at least 1 prescription claim for saxagliptin or sitagliptin between January 1, 2010, and December 31, 2011, at least 6 months of continuous eligibility before treatment initiation (baseline period), and at least 6 months of continuous eligibility after treatment initiation (follow-up period).

Patients with a claim for saxagliptin or sitagliptin during the baseline period, and patients who initiated multiple index therapies (multiple claims of DPP4 inhibitors at the index period), were excluded from the study. Patients were excluded if they were found to have both saxagliptin and sitagliptin at index (treatment initiation period). Combined therapy at initation was not allowed.

We used intention-to-treat design and our patient selection was based on baseline information on saxagliptin and sitagliptin. As a result, we did not exclude patients that switched medications. Our goal was to compare patients that are initiated on saxagliptin with those who are initiated on sitagliptin.

### Study variables

The outcome variables included charges in the individual 6-month follow-up period. These computed charges were comprised of overall charges (i.e., both medical and pharmacy claims), overall medical charges, and overall pharmacy charges. The overall charges were the sum of medical and pharmacy costs paid by health plans and patients’ out-of-pockets for all medical and pharmacy claims for the 6-month follow-up period. Overall medical charges included non-prescription medical claims, while overall pharmacy charges included the total of prescription costs.

Diabetes-specific charges, such as diabetes-related overall charges (i.e., both medical and pharmacy claims), diabetes-related medical charges (calculated from inpatient and outpatient claims related to diabetes), and diabetes-related pharmacy charges, were assigned. The claims data provided information on ICD-9 CM codes associated with these charges. Based on medical claims with a primary or secondary ICD-9 CM diagnosis code for T2D and prescription claims for antidiabetes medications. Hospitalization rates in the 6-month follow-up period were also determined; these rates included overall inpatient hospitalization rates and diabetes-related inpatient hospitalization rates.

The baseline variables included the following: demographics (i.e., age, gender, and index year); comorbid conditions in the baseline period (i.e., cardiovascular events, obesity, dyslipidemia, stroke, atherosclerosis, retinopathy, hypertension, nephropathy, diabetic foot ulcer, neurologic complications, dental disease, and renal impairment); comorbid indices (i.e., Charlson Comorbidity Index [CCI], Diabetes Complications Severity Index [DCSI8] Dichotomized as 1–4 and >5, and Psychiatry Diagnostic Groups [PDGs7], which assessed whether a patient belonged to any of the 12 PDG categories); pharmacotherapy - use of any antidiabetes medication in the baseline period (yes/no); number of classes of antidiabetes medications used in the baseline period; polytherapy at index with specific antidiabetes medications biguanides, thiazolidinediones, sulfonylureas, insulin, or other polytherapy; use of other medications in the baseline period - antihypertensives and statins; and baseline resource utilization – copay (co-payment) value for index drug at index date (copay of the studied drugs at baseline period), inpatient hospitalization, and overall charges.

### Statistical analysis

Propensity score matching was used to balance the cohorts of patients prescribed saxagliptin or sitagliptin. Propensity scores predicting use of saxagliptin or sitagliptin were generated using multivariate logistic regression based on demographic characteristics, comorbidities in the baseline period, comorbidity indices, baseline resources use, and baseline pharmacotherapy before the index date. Certain variables such as DCSI were categorical, while CCI was matched as continuous variable. Propensity score matching aims to adjust the selection bias in nonexperimental, nonrandomized, and retrospective observational studies. It allows the mirroring of each patient in the saxagliptin cohort with a patient with similar characteristics in the sitagliptin cohort. Based on the balancing guidelines [17], a 1:1 propensity-matched sample of saxagliptin and sitagliptin patients produced cohorts with more balanced baseline characteristics. Hence, a 1:1 caliper-matching technique was created to reduce any potential confounding. The result was that the patients were matched using the calipers of width equal to 0.01 of the standard deviation of the logit of the propensity score. Although there are no specific guidelines for the size of caliper to be used, it is advised to use a tighter caliper to reduce the bias. The study picked 0.01 caliper because it gave us maximum closeness along the variables while maintaining the appropriate sample size.

After matching, statistical tests were conducted to compare saxaglipitin initiaters with sitaglipitin initiators. Variables that were normally distributed such as healthcare utilization and other independent variables were compared using t-tests and chi-square tests However, for cost adjusted values, a univariate generalized linear model (GLM) with a log link function and γ distribution for the error term was used to account for the non-normal distributions associated with cost data . All outcomes were assessed based on an intent-to-treat analysis in the 6-month follow-up period. Statistical significance was evaluated at *P* < 0.05. Analysis were conducted with SAS version 9.3 (SAS Institute Inc. Cary, NC, USA).

## Results

**Fig. 1:**
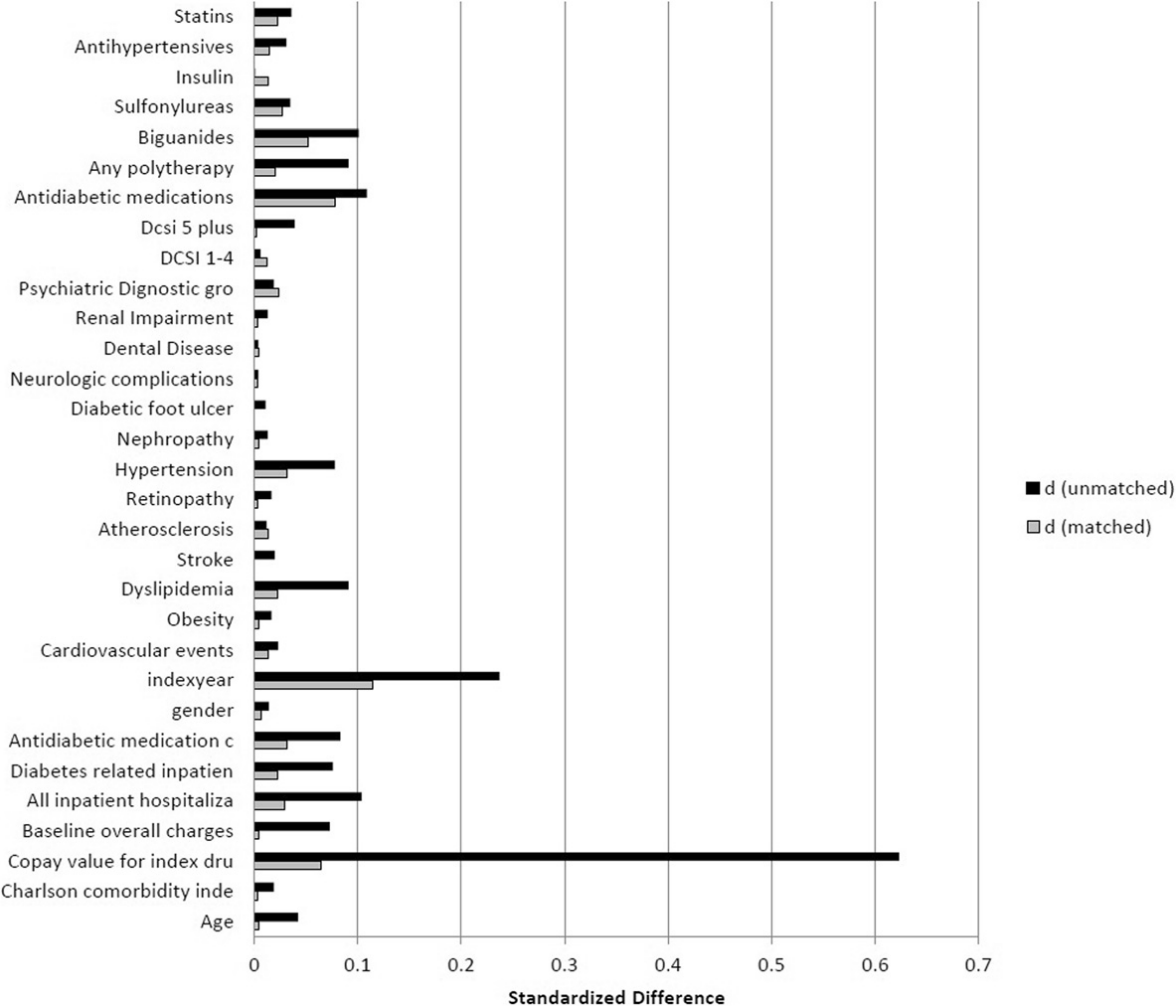
Covariate balance

Data from 31,503 patients were used for the analyses. Patients were divided into two cohorts each receiving saxagliptin or sitagliptin treatment in the 6-month follow-up period. The cohorts were balanced using the propensity score matching technique; after propensity score matching each cohort had 7711 patients, and no significant differences were observed in most of the above mentioned baseline characteristics of demographics, comorbid conditions, comorbid indices, pharmacotherapy, polytherapy, use of other medications in the baseline period, disease severity proxy, and baseline resource utilization (Table 1). However, index year, hypertension comorbidity, copay at index date, use of biguanides, taking antidiabetes medication at baseline, and number of antidiabetes medication classes were still significantly difference between two groups; but the differences are smaller compared to the pre-matching differences. Propensity score matching reduced the differences under 15 %. In case of remaining variables, propensity matching helped bring standardized differences much closer to 0, indicating the improved covariate balance (Fig. 1).

**Table 1:** Baseline characteristics of patients who initiated treatment with saxagliptin or sitagliptin

	Before propensity matching			After propensity matching		
	Saxagliptin (*n* = 8438)	Sitagliptin (*n* = 23,155)	P value	Saxagliptin (*n* = 7711)	Sitagliptin (*n* = 7711)	P value
Demographics						
Age, years, mean (SD)	54.4 (10.0)	54.8 (10.6)	0.001	54.53 (9.96)	54.58 (10.60)	0.7590
Men, %	56.6	55.9	0.265	56.28	56.63	0.6610
Index year, %			<0.001			0.0132
2010	43.0	54.8		45.13	39.49	
2011	57.0	45.2		54.87	60.51	
Comorbidities, %						
Cardiovascular disease	20.3	21.2	0.067	20.50	19.98	0.4228
Obesity	10.5	11.1	0.194	10.58	10.47	0.8133
Dyslipidemia	71.9	67.7	<0.001	71.40	72.45	0.1466
Stroke	2.9	3.3	0.126	3.00	3.00	1.000
Atherosclerosis	1.9	2.1	0.337	1.92	1.74	0.4001
Retinopathy	4.3	4.6	0.189	4.45	4.38	0.8446
Hypertension	69.7	66.1	<0.001	68.88	70.35	0.0459
Nephropathy	2.5	2.7	0.305	2.48	2.54	0.7969
Diabetic foot problems	0	0	0.432	0.03	0.03	1.000
Neurological complications	5.5	5.4	0.763	5.38	5.45	0.8588
Dental disease	0.1	0.1	0.754	0.08	0.06	0.7629
Renal impairment	6.9	7.3	0.315	6.87	6.94	0.8738
Psychiatry diagnostic group	11.0	11.6	0.147	11.18	10.43	0.1324
DCSI 1–4	27.4	27.1	0.624	27.45	26.91	0.624
DCSI ≥5	4.7	5.5	0.002	4.72	4.67	0.8790
CCI, mean (SD)	1.66 (1.35)	1.69 (1.49)	<0.05	1.66 (1.35)	1.67 (1.40)	0.8292
Baseline resource use						
Copay value for index drug at index date, mean (SD)	55.3 (34.4)	35.9 (27.4)	<0.001	52.57 (32.64)	50.39 (33.88)	<0.0001
Baseline overall charges (%)			<0.001			0.7638
Quartile 1 (lowest charges)	17.7	19.2		17.66	16.95	
Quartile 2	28.2	26.4		28.10	28.69	
Quartile 3	30.4	28.3		30.14	30.93	
Quartile 4 (highest charges)	23.7	26.1		24.10	23.43	
Inpatient Hospitalizations, %						
All	6.7	9.5	<0.001	7.15	6.42	0.0729
Diabetes related	4.6	6.8	<0.001	4.86	4.50	0.4785
Baseline Pharmacotherapy						
Antidiabetic medications, % yes	75.9	72.1	<0.001	75.70	77.46	0.0097
Antidiabetic medication classes, mean (SD)	1.32 (1.04)	1.23 (1.03)	<0.001	1.31 (1.04)	1.34 (1.02)	0.0476
Polytherapy, %						
Any Polytherapy	56.3	60.8	<0.001	56.45	55.48	0.2238
Biguanides	48.4	53.5	<0.001	48.74	46.12	0.0011
Sulphonylureas	12.5	11.3	0.006	12.27	13.18	0.0907
Insulin	4.2	4.2	0.949	4.16	4.44	0.4045
Other Medications, %						
Antihypertensives	68.3	66.8	0.014	67.95	68.64	0.3590
Statins	49.3	47.5	0.005	49.02	50.12	0.1710

Saxagliptin treatment was associated with lower overall charges ($13,292 vs $14,032; *P* = 0.0023) and overall medical charges ($9,540 vs $10,296; *P* = 0.0024) during the 6-month follow-up period compared with sitagliptin treatment.

**Fig. 2:**
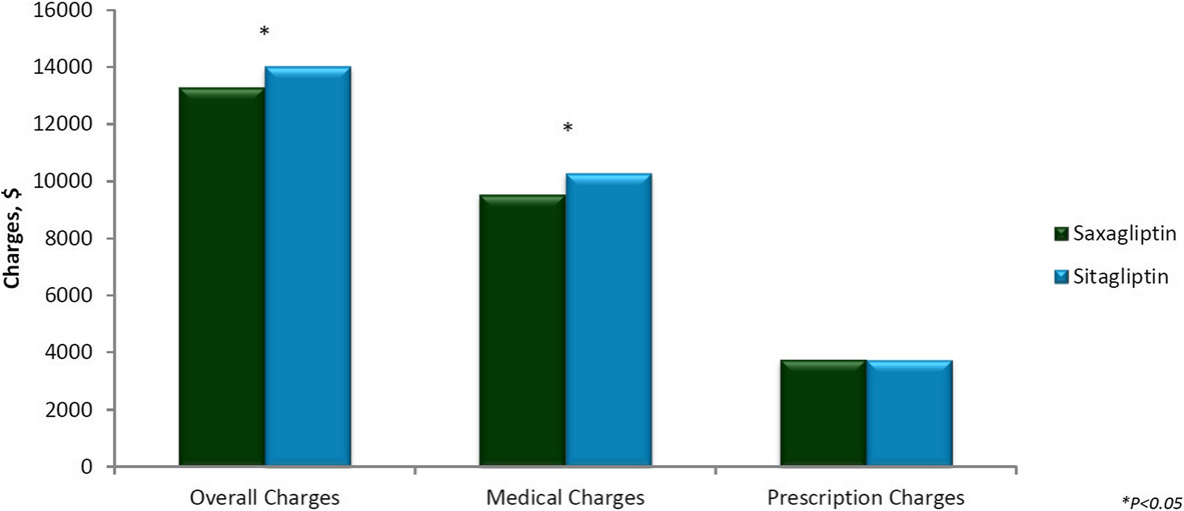
Mean overall healthcare charges during the follow-up period: after propensity matching

Pharmacy charges did not differ significantly between saxagliptin treatment and sitagliptin treatment ($3,751 vs $3,735, *P* = 0.6937, respectively) (Fig. 2).

**Fig. 3:**
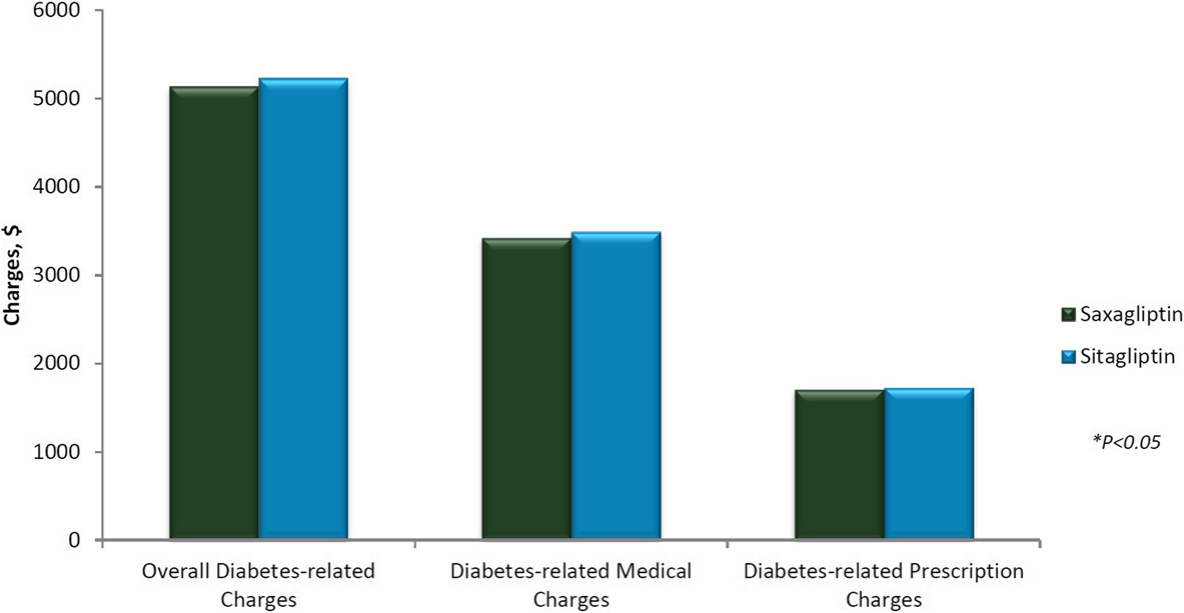
Mean diabetes-related healthcare charges during the follow-up period: after propensity matching

No significant differences were associated with diabetes-related overall charges ($5,141 vs $5,232; *P* = 0.2957), diabetes-related medical charges ($3,428 vs $3,499; *P* = 0.4998), and diabetes-related pharmacy charges between saxagliptin treatment and sitagliptin treatment ($1,713 vs $1,732, *P* = 0.2495, respectively) (Fig. 3).

**Fig. 4:**
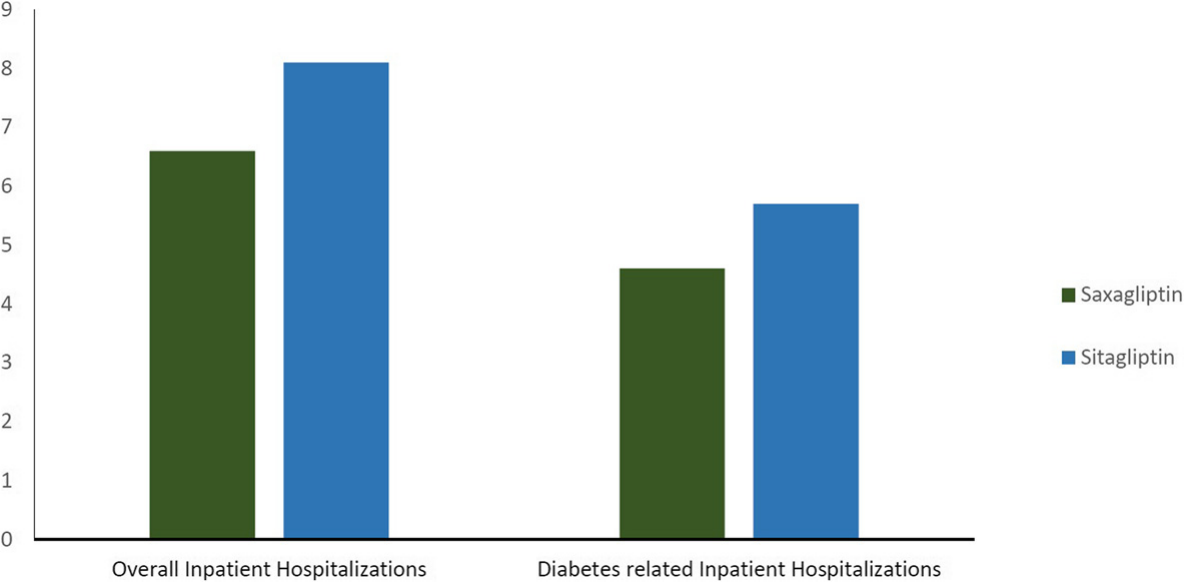
Inpatient hospitalization rates during the follow-up period: after propensity matching

Inpatient hospitalization rates were significantly lower with saxagliptin treatment than with sitagliptin treatment in terms of all-cause hospitalizations (6.6 % vs 8.1 %; *P* = 0.0001) and diabetes-related hospitalizations (4.67 % vs 5.78 %; *P* = 0.0019) (Fig. 4). All-cause inpatient charges and diabetes-related inpatient charges were significantly lower with saxagliptin ($2,917.26 vs $3,445.89; *P* < 0.0001 and $1,325.13 vs $1,342.12; *P* = 0.0003, respectively).

## Discussion

The total healthcare costs attributable to T2D are substantial. Medical costs attributed to T2D include $27 billion for direct care, $58 billion to treat patients with diabetes-related chronic complications, and $31 billion in excess general medical costs. Given the significant economic burden of T2D, reducing it is of major significance to payers. Therefore, it is in the best interests of healthcare payers to adopt the most cost -saving treatment strategies that would yield maximum benefits at minimum cost. This study was designed to inform healthcare decisions by comparing resource utilization over a 6-month period following initiation of one of 2 commonly prescribed DPP4 inhibitors in a real world setting using insurance claims data in the US.

Research has suggested that sitagliptin and saxagliptin both produce similar improvements on HbA1c levels, from 0.5 to 1.2 %, with no increase in the risk of hypoglycemia unless combined with other therapies [15, 18, 19]. Assuming this clinical equivalence, other factors such as the ease of dosing among patients with renal impairment, tolerability, lower copays, or drug-drug interactions might lead to a difference in the real world cost and resource utilization between saxagliptin and sitagliptin. In support of a possible difference in ease-of-use, Farr et al. showed that saxagliptin is associated with greater adherence compared with sitagliptin, which could be due to one-step adjustment with saxagliptin in renally impaired patients, instead of two-step adjustment with sitagliptin [18]. Interestingly, our study also found that there was a small, but significant, difference in the copays at the baseline between saxagliptin and sitagliptin groups (the average difference was $2.18). Thus, it is important to determine whether these factors would result in the difference in real world resource utilization between saxagliptin and sitagliptin.

Our analysis shows that lower overall costs and inpatient utilization within a 6-month follow-up period were observed in patients initiating saxagliptin treatment. Overall charges, overall medical charges, and inpatient charges and utilization were significantly lower in patients initiating saxagliptin treatment. Higher inpatient utilization with sitagliptin might have contributed to higher medical charges and higher overall charges. This is consistent with the finding of prior research using a different database and matching method [16]. However, diabetes-related charges did not significantly differ between saxagliptin and sitagliptin users in our study. This might be due to the difficulty in determining whether a particular complication is related to diabetes by clinicians. For example, diabetes increases the risk of many severe cardiovascular complications. However, these complications of diabetes can occur among patients without diabetes, and it is difficult for clinicians to determine if a particular complication is attributable to diabetes. Consequently, the classification of diabetes-related complications is imperfect.

Using the Januvia Diabetes Economic Model (a discrete event simulation model for long-term outcomes of diabetes), Schwarz et al. has found sitagliptin to be more cost-effective than thiazolidinediones and sulfonylureas even with a high cost per day [19]. These results suggest cost-effectiveness of DPP4 inhibitors compared with other commonly available oral medications. saxagliptin also belonging to the same category of DPP4 inhibitors, results from Januvia Diabetes Economic model can be extrapolated to demonstrate potential cost effectiveness of saxagliptin in a similar way. Future research needs to study and confirm these findings by conducting a cost-effectiveness analysis of saxagliptin compared to other antidiabetes oral medications. It should be also noted that this analysis is carried out using insurance claims data from a US population and may not be generalized to other countries as the healthcare system in different countries may differ with respect to payment system and formulary structure.

## Limitations

Due to selection bias, propensity score matching was used to reduce confounding from differences in the patients’ baseline characteristics. While the majority of baseline variables became indifferent between patients taking saxagliptin and sitagliptin after matching, few variables such as index year were still significantly different between two groups (*p* < 0.05). However, these differences were reduced after matching when compared to those before matching, Additionally, we cannot rule out the possibility of residual confounding, particularly due to differences in duration of disease or severity (e.g., HbA1c), which was not available in this database. Because clinicians likely initiate saxagliptin and sitagliptin in patients with similar disease duration, and the baseline hospitalization rates post matching do not significantly differ between the saxagliptin and sitagliptin groups, the possibility of selection bias is limited. The data may fail to generalize well to other populations. Although the intent to treat design provides several advantages it also adds a few limitations. One such limitation is difference in end-point due to a large proportion of participants cross over to opposite treatment arms However, in this analysis the follow up period being 6 months such risk is a minimum. In addition Intent to treat analysis has been criticized for being too cautious and thus being more susceptible to type II error [20, 21].

## Conclusion

Compared with patients initiating sitagliptin treatment, patients initiating saxagliptin treatment reported lower overall and medical charges and lower overall and diabetes-related hospitalization rates. These findings may aid payers in managing patients with T2D.
